# Information asymmetry and deception

**DOI:** 10.3389/fnbeh.2015.00109

**Published:** 2015-07-21

**Authors:** Irma Clots-Figueras, Roberto Hernán-González, Praveen Kujal

**Affiliations:** ^1^Department of Economics, Universidad Carlos III de MadridSpain; ^2^Division of Industrial Economics and Finance, Business School, University of NottinghamNottingham, UK; ^3^Economics, Business School, Middlesex UniversityLondon, UK

**Keywords:** investment game, asymmetric information, deception, understatement, overstatement

## Abstract

Situations such as an entrepreneur overstating a project's value, or a superior choosing to under or overstate the gains from a project to a subordinate are common and may result in acts of deception. In this paper we modify the standard investment game in the economics literature to study the nature of deception. In this game a trustor (investor) can send a given amount of money to a trustee (or investee). The amount received is multiplied by a certain amount, *k*, and the investee then decides on how to divide the total amount received. In our modified game the information on the multiplier, *k*, is known only to the investee and she can send a non-binding message to the investor regarding its value. We find that 66% of the investees send false messages with both under and over, statement being observed. Investors are naive and almost half of them believe the message received. We find greater lying when the distribution of the multiplier is unknown by the investors than when they know the distribution. Further, messages make beliefs about the multiplier more pessimistic when the investors know the distribution of the multiplier, while the opposite is true when they do not know the distribution.

## Introduction

Lying (or deception) in Economics is a rational act if it leads to an increase in one's payoffs. Recent research has shown there may be several motives to lying ranging from pure selfish to altruistic (Gneezy, 2005). Meanwhile, there is a vast literature on the neuro-biological basis of lying and manipulative behavior. Zhu et al. (2014) state that substantial correlational evidence suggests that prefrontal regions are critical to honest and dishonest behavior. For example, Yang et al. (2005) studied prefrontal gray and white matter volumes for 12 individuals who pathologically lied, cheated and deceived. They found that liars showed a 22–26% increase in prefrontal white matter and a 36–42% reduction in prefrontal gray/white ratios relative to their controls. It has been shown that the dorsolateral prefrontal cortex affects tradeoffs between honesty and self-interest (Zhu et al., 2014) and that there may also be gender differences in neural basis of deception (Marchewka et al., 2012).

Lying is a prevalent daily phenomenon and is a part of daily social interaction. Importantly, lying behavior has important economic consequences. It is also for this reason that the study of lying is important. While the neuro-biological literature has studied the neurological underpinnings of deception, experiments in the economics literature have studied how and when individuals lie in incentivized settings. Starting with Gneezy (2005), the recent literature in experimental economics has studied specifically the nature of lies for a randomly drawn sample of University students. Using incentivized experiments this literature has found that lying is prevalent, may not always be selfish, and may have many motivations.

This experimental literature has classified the nature of lies based upon the gains and losses they impose on a third party. For example, Gneezy (2005) defines four categories of lies. The first category is composed by lies that help both sides, or at least do not harm anyone (white lies). The second are lies that may benefit the other person even though they may harm the liar. The motivation for such lies may be altruism or efficiency motives. Thirdly, a lie may not help the liar but may harm at least one person (if not both). These would be classified as spiteful lies. Finally, Gneezy studies lies that decrease the payoff to the other party and increase the payoff to the liar; these are classified as black lies. He finds that people are sensitive to the harm their actions may cause when deciding to lie. In addition, he finds that this unselfish motive diminishes with the size of the gain to the decision maker. Sanchez-Pages and Vorstaz (2007) investigate why a majority of subjects tell the truth when incentives would suggest otherwise. They find a group of subjects with preferences for truth-telling and one that only cares about material incentives. Hurkens and Kartik (2009) modify the game in Gneezy (2005) and find that there is lying aversion but that results are also consistent with the fact that some people never lie and others lie only in their own benefit. Erat and Gneezy (2013) study white lies (altruistic or Pareto improving) and find that there is reluctance to tell Pareto improving lies indicating pure lying aversion.

There are other lies that do not rely on the gains and losses from the outcomes. In this case the perception that one transmits may be more important. For example, (Fischbacher and Föllmi-Heusi, 2013) find that people may lie to not appear dishonest and lies may even be disadvantageous to one-self.^1^ Utikal and Fischbacher (2013) study disadvantageous lies where individuals may lie to maintain a positive self-perception. They argue that if the utility gained from maintaining the perception outweighs the monetary cost then people will tell disadvantageous lies. Sutter (2009), meanwhile, studies how truth telling can be a strategic form of lying when the truthful message may not be believed. In addition, Hao and Houser (2013) show that people may also have preferences on lies and may prefer incomplete lies over bigger ones. Finally, Kriss et al. (2013) study deception in an ultimatum bargaining game under asymmetric information. They find that deception is greater when informed parties make an explicit statement than when information is communicated implicitly. This is especially true for larger stakes. However, allowing the explicit statement to be accompanied by a promise of truthfulness reverses this effect. Finally, as in our paper they also observe very high levels of dishonesty.

Besides the taxonomy of lies mentioned above, there are many real life situations that involve over, and under, statement of the truth. Think of a typical situation in a firm where a superior proposes her subordinate to work on a project. Clearly, the superior has additional knowledge about it and knows the potential gains it entails for himself and the subordinate, and may choose to over (or under) sell it. Another example is venture capital where the investee may oversell a project to make it look attractive^2^. In these examples the information transmitted regarding the return on an investment may not be verified easily and can have adverse economic (efficiency) consequences.^3^ Situations with asymmetric information are pervasive and may result in deception due to conflict of interests.

We introduce information asymmetry and communication in a modification of the standard investment game (Berg et al., 1995). In this game, two players, an investor and an investee, are endowed with an initial amount of money. The investor must decide how much of his initial endowment to invest. The amount invested is multiplied by a parameter (the multiplier) greater than one. Then, the investee can decide how much of this amount, if any, is returned to the investor, keeping the remaining amount for herself. We introduce information asymmetry in this setting by informing the investee about the true value of the multiplier, whereas the investor only has information about the distribution of the multiplier. We introduce communication by allowing the investee to send a numerical message to the investor regarding the value of the multiplier. The investee can decide whether to inform the investor truthfully, or not. The possibility of communication between the investee and the investor is what allows us to study the nature of messages and deception.

We find that under-, and over-, statement of messages is common with a large proportion of subjects sending false messages. We find that almost half the investors believe the message they receive. Further, we find that messages understating the true value are more likely to be believed.

We also explore why investees may decide to deceive investors by understating or overstating the true value of the multiplier. We analyze some possible explanations from the literature that may explain lying through under, or over, statement. Investees may want to overstate (the true value of *k*) if investors are naive, or believed to be so, and their investment is expected to increase with the value of *k*. In this regard, our structure has the flavor of the cheap-talk scenario in Ottaviani and Squintani (2006) and Kartik et al. (2006)^4^ where message inflation will be observed if those who send the message believe that the receivers of the message are naive. We find evidence of this behavior as those investees who overstate the true value of *k* expect to receive greater investments than those who tell the truth or understate it.

While overstatement may occur if investors are considered naive, there are several competing explanations for message deflation. It may be that investees have beliefs regarding what types of messages are believable (Sutter, 2009; Fischbacher and Föllmi-Heusi, 2013). Another possible explanation is guilt aversion (Charness and Dufwenberg, 2006, 2010). Investees may understate the true value of *k* if they expect to return a low amount and therefore feel less guilty. Finally, individuals may have preferences over the degree and the direction of the lies. In this scenario individuals may view small (i.e., partial) lies as more acceptable than big ones (see Hao and Houser, 2013)^5^, and understatement could be perceived as more acceptable than overstatement. We find evidence for all of these possible explanations.

In our paper the investees can lie to obtain the maximum benefit for themselves (a black lie), but they can also lie to maximize the total surplus, by increasing the amount sent by the investor, and benefit later both parties (a white lie). Therefore, lies can have positive or negative consequences to both parties depending on the posterior investee's decision. In this regard, we can consider them as gray lies and fit them between white and black lies in Gneezy's classification. Further, in our structure lies are endogenous and may lead to under, and over, statement.

Though the game they study is different, the two papers closest to ours are Gneezy (2005) and Fischbacher and Föllmi-Heusi (2013). In Gneezy (2005) subjects have 2 options: lie or tell the truth, in our case they report a numeric message so lying is endogenous and there can be different degrees of lying. This is more similar to real-life situations in which the information given is, for example, the returns on an investment, or the probability that a project succeeds. As in our paper, lying in Fischbacher and Föllmi-Heusi (2013) is endogenous. Subjects can over or under state the true values taken by the roll of a dice. In their design the direction of the lie determines the payoffs. The difference between Fischbacher and Föllmi-Heusi (2013) and this paper is that in our scenario the consequences of the lies are also endogenous, given that the investor can react in different ways to the message and send a larger or smaller fraction to the investee, who can then decide how much to return.

We contribute to this literature by studying a message that is open to interpretation by the receivers of the message. The lies have a strategic element to it as they seek to exploit knowledge regarding the beliefs. We also analyse the effect of ambiguity on the distribution of the true state, i.e., the value of the multiplier which, to the best of our knowledge, has not been analyzed before. Ambiguity is all pervasive in our daily interactions. In our everyday interactions we frequently encounter situations where risk is not quantifiable (i.e., ambiguous), while situations with defined probabilistic outcomes are few. For example, future stock prices are ambiguous^6^, as is the quality of education, or the outcome of a business venture (as we state in the venture capital example earlier). We find that introducing ambiguity may exacerbate the effect of information asymmetry as we observe higher levels of deception.

## Methods

Anonymity was always preserved (in agreement with Spanish Law 15/1999 on Personal Data Protection). No association was ever made between participants' names and the results. As is standard in socio-economic experiments, no ethic concerns are involved other than preserving the anonymity of participants. This procedure was checked and approved by the Deans at the University Carlos III of Madrid; the institution hosting the experiments. At that time no official IRB was established at the university.

### Participants and general protocol

A total of 694 undergraduate students from the university were recruited for an hour and the average payoff was approximately €20. Including the instructions, the experiment lasted approximately 45 min. Prior to their recruitment, all subjects were given a questionnaire (see supplementary SI1). Responding to the questionnaire was a pre-requisite to participating in the experiments. The questionnaire contained personal information about age, studies, grades, family origin etc.

All instructions were computer based (see supplementary SI2). This protocol was implemented to ensure strict anonymity across subjects and to minimize the interaction with the experimenter. After making their decisions subjects responded to a set of questions (see supplementary SI3). The experiment ended after all subjects had responded to this second set of questions. They were then called out individually and paid their earnings privately.

### The investment game

Subjects played the investment game introduced by Berg et al. (1995). In this game, two individuals, investee and investor, receive an initial endowment of 100 dex (experimental money; approx. $12). The investor can send any amount (M) between 0 and 100 dex to the investee. The amount sent by the investor is then multiplied by *k* (the multiplier). Finally, the investee decides how much of the amount received (*k* × *M*) is returned to the investor, keeping the remaining amount for herself.

Individuals were randomly selected into sessions and roles were randomly assigned. Investees and investors were assigned to separate rooms in the same building before they arrived for the experiment and were referred to as player A and player B, respectively. Both were told they would be paired with another person (A/B) in a different location.

### Treatments

We compare variations of the standard investment game (Berg et al., 1995) where we introduce uncertainty (for the investor) regarding the value of *k*. We introduce information asymmetry by giving the information on the value *k* takes only to the investee. From the beginning, the investee gets to know the actual value of *k*, and knows that the investor does not know this value. All this is common knowledge for both players. Further, in these treatments we also allow for communication between the subjects. The investee can decide whether to send a numerical message informing the investor about the value of k.^7^ The investee is free to state any value of *k* and may choose to deceive the investor or not.

Below we describe the main treatments in detail and then provide justification for the treatment selection. The treatments are also summarized in Table 1.

**Table 1:** **Treatments**.

		**Timing of the messages**		
		**No messages**	**Message before investor's decision**	**Message after investor's decision**
Information known by the investor about *k*	*k* ∈ {2, 3, 4}	234_No	234_ExAnte	234_ExPost
	k > 1	*k*>1_No	*k*>1_ExAnte	

#### Treatment 234_No *k* (= *2, 3 or 4*)

The investor is told that *k* can now take any value between {2, 3, 4} with equal probability.

#### Treatment 234_ExAnte

Ex-ante message about *k (= 2, 3, or 4)* The only difference from treatment 234_No is that now the investee can decide whether to send a message or not regarding the value of *k* prior to the decision of the investor. The message is numerical and the investee can input any value. Upon receiving the message the investor decides how much to send to the investee. In case of no message the investor is informed that there is no message.

In order to further analyse the reasons why individuals over, or under, state the value of *k* we conducted another treatment in which the investees send a message ex-post:

#### Treatment 234_ExPost

Ex-post message about *k* (= *2, 3, or 4*) The only difference from treatment 234_ExAnte is that the investee informs (or not) the investor of the value of the *k* after the investor has decided how much to send.

Finally, we study how the degree of information asymmetry affects false messages. In order to do this, we conducted two additional treatments where the investor was not informed about the distribution of *k*.

#### Treatment k>1_No

Ambiguity treatment The only difference from the 234_No treatment is that the investor is told that *k* can now take any value greater than one. From the beginning, the investee gets to know that the value of *k* is equal to 3, and is told about the information the investor has. All this is common information.

#### Treatment k>1_ExAnte

Ex-ante message about *k (> 1)* The only difference between treatment *k*>1_No and this treatment is that now the investee can send a (numerical) message indicating the value of *k* prior to the investor's decision.

The nature of pre-play communication we have is minimal. Given the value of the return on the investment (*k*), the investee can send any numeric announcement to the investor regarding the value *k* takes^8^. While compromising on the richness of language,^9^ using only numeric values has an advantage as it enables us to clearly measure the direction and the size of the false message, i.e., above or below the actual value. This also enables us to measure clearly when subjects over-, and under-, state in their messages. We further avoid the difficulties associated with the subjective classification of language. The use of language may be an important factor in reciprocity if agents have preferences on “language.” That is, agents may interpret language differently and respond accordingly to it. Note, however, that the study of reciprocity is not the focus of this paper.

## Results

In this section we first study the nature of false messages, i.e., how subjects inflate, or deflate, messages. We then study whether investors believe the messages they receive. Finally, we analyze subjects' behavior in the game and their responses to the questionnaire.

### False messages

In principle any message sent should be interpreted as cheap talk. However, if investees believe that investors are naive, i.e., believe the messages they receive, they may be interested in sending a message to influence their investment decision. In this case, if investees believe that the amount sent by the investors may increase with the return on the investment, *k*, they may then overstate their messages in order to receive a higher amount from the investor (Ottaviani and Squintani, 2006). Contrarily, the investees may decide to understate the value of *k* if they anticipate that they will return a very small amount to the investor. In other words, understating *k* and subsequently returning less can be explained by guilt aversion (Charness and Dufwenberg, 2006), where decision makers feel guilty if they disappoint others. If this is the case then messages can influence what the investor will expect to receive, and to avoid disappointment, investees might report a lower value of *k* if they expect that they will return little.^10^ Note that investees could believe that lower values of *k* are more believable, and they could then decide to understate *k*.

**Figure 1:**
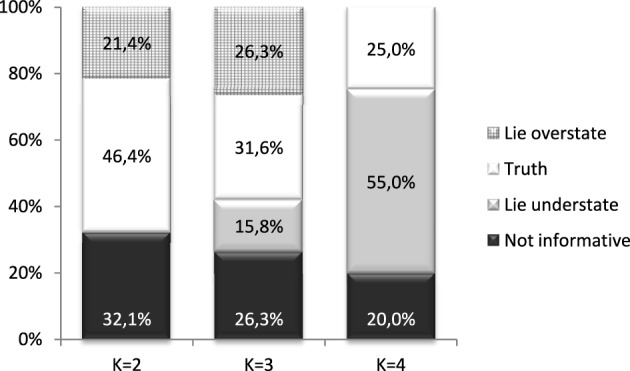
**Distribution of messages by return**. Treatment is 234_ExAnte.

We will now look at results from the treatment (234_ExAnte) where *k* can take any value from the distribution {2, 3, 4} with equal probability. Investees can choose to send a message, or none, regarding the value of *k*. We have data from 134 students, organized in 67 pairs. First we identify those who sent false messages and those who told the truth (see Figure 1). Of all investees in this treatment, 14.93% decided not to send any message while, 11.94% sent a message outside of the known distribution. We consider these two groups as sending an uninformative message. Investees may choose not to send a message due to two reasons. One may be that they think that any message they send may be considered as “cheap talk.” On the other hand a message may not be sent to not transmit any information to the investor.^11^

Excluding the subjects who sent an uninformative message, almost the same proportion of individuals sent false messages, 51.02%, and told the truth about the value of *k*, 48.98%. Meanwhile, 28.57% understated *k*, while 22.45% overstated it.

We classify false messages according to the difference between the real and the reported values of *k* for each value of *k* (Figure 1). The proportion of individuals who told the truth is higher for low values of *k*: 46% (32%) [25%] when *k* took value of 2 (3) [4]. However, pairwise tests of proportions (Table 2) indicate that the differences in proportions are not statistically significant. The proportion of individuals acting strategically and overstating the true value of *k* is the same when *k* took the value of 2 or 3. Understatement of *k* is significantly higher for *k* = *4* than when *k* = *3*.

**Table 2:** **False messages**.

	**Tests of proportions (*p*-value)**		
	***k* = 2 vs. *k* = 3**	***k* = 2 vs. *k* = 4**	***k* = 3 vs. *k* = 4**
Told the truth about *k* (*k* = message)	0.3086	0.1305	0.6481
Overstated the value of *k* (*k* < message)	0.6976	–	–
Understated the value of *k* (*k* > message)	–	–	0.0402

*Treatment is 234_ExAnte*.

We may get understatement due to two reasons. First, and consistent with the guilt aversion hypothesis, investees may believe that the larger the reported value of *k* the more investors are going to be likely to believe that they will be returned a large amount. As a result, if they plan on returning a small amount, they will be more likely to understate the value of *k* when *k* is high. The second reason could be strategic. If investors are not naive and have prior beliefs on subject responses, investees may strategically best respond. In this case investees may strategically understate to make their messages believable, and to induce investors to send a larger amount. In the following section we will see that investors find smaller values more believable than large ones.

We now study whether our results are robust to the introduction of ambiguity regarding the value that *k* can take. Most situations in real life involve a certain degree of ambiguity, for example, situations with defined probabilistic outcomes are very few. We want to analyse whether lying behavior is similar under ambiguity. Similarly, Rode (2010) varies the level of information asymmetry to study the effect on lying and trust. He finds that advisors tell the truth more frequently when decision makers' uncertainty is low, as advisors feel less morally bound to send accurate information. Decision makers followed the recommendations independently of the level of uncertainty. However, Rode (2010) finds that inducing a competitive environment, previous to the communication game, reduced the proportion of recommendations that are followed by the decision makers when the level of uncertainty is low or medium.

**Figure 2:**
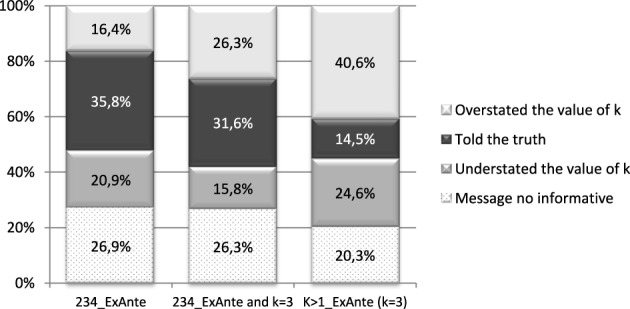
**Distribution of messages by return and treatment**.

We compare the results of the uncertainty treatment (234_ExAnte) with those of the ambiguity treatment (*k* > 1_ExAnte). Contrary to Rode (2010), we find that the proportion of subjects who tell the truth in the ambiguity treatment is significantly smaller while, the proportion of the subjects who overstate the value of *k* is higher (see Table 3 and Figure 2). This can be partially due to the fact that *k* is always equal to 3 in the ambiguity treatment. When comparing the subset of subjects in treatment 234_ExAnte for which *k* = 3 to those in the ambiguity treatment (*k* = 3), the proportion of subjects who tell the truth is much smaller in the latter, but the proportion of subjects overstating returns are not significantly different. This shows that the introduction of ambiguity encourages false messages.

**Table 3:** **False messages in the ambiguity treatment**.

	**Tests of proportions (*p*-value)**	
	**234_ExAnte vs. *k* > 1_ExAnte**	**234_ExAnte vs. *k* > 1_ExAnte (*k* = 3 in both cases)**
Told the truth (*k* = message)	0.0041	0.0872
Overstated the value of *k* (*k* < message)	0.0018	0.2556
Understated the value of *k* (*k* > message)	0.6032	0.4150

### Are messages believable?

We obtain information on subject beliefs from the questionnaires we conducted after the players made their decisions. This allows us to see whether messages are believed. This information is useful as it also tells us whether investors are naive.

In the questionnaire we asked investors about the value they thought *k* took.^12^ Considering only those pairs in which the investee sent an informative message, a sizeable proportion of investors (45.16%) believed the message, while 48.39% of the investors believed that the investees were overstating the value of *k*. In contrast, only 6.45% believed that investees understated the value of *k*.^13^ This means that most investors expected the investees to act strategically and to overstate *k*.

Overstating k did indeed induce subjects to send a larger amount. We check whether those investors who believed that *k* took larger values sent a larger amount. In order to do this, we compare investors with different beliefs. In the treatment 234_ExAnte, those investors who believed (as reported in the questionnaire) that *k* = 2 sent on average (median) 33.5 (25.0), while those who believed *k* = 3 sent 59.0 (55.0). This difference is significant (Mann–Whitney *p* = 0.0055, *t*-test *p* = 0.0145, and the median test *p* = 0.016). If we consider all treatments with *k* = {2,3,4} (234_No, 234_ExAnte, 234_ExPost), investors who believed *k* = 2 sent on average (median) 36.0 (30.0), while those who believed *k* = 3 sent 53.6 (60.0), with Mann–Whitney *p* = 0.0023, *t*-test *p* = 0.0028 and Median test *p*<0.001. We do not have enough data to do the same analysis for those who believed *k* = 4 (only 9 observations for the three treatments)^14^.

Interestingly investors were more likely to believe the message when the reported value of *k* was low, i.e., 89% (36%) [25%] of the investors believed a message stating that *k* was equal to 2 (3) [4]. This suggests that if investors are not naive then investees may also have incentives to understate *k*. That is, there is also a strategic motive to understate *k* to make messages more credible.

**Figure 3:**
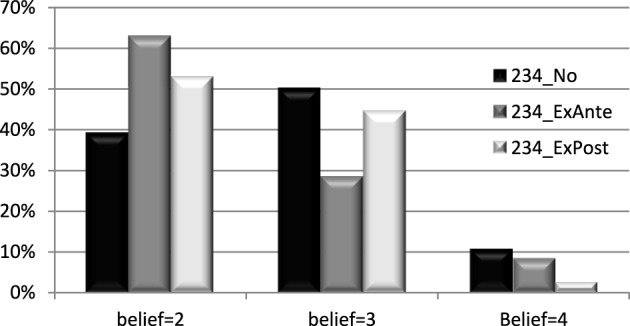
**Distribution of beliefs by treatment**. Note: Proportion of subjects by treatment who believed that the value of *k* was 2, 3, or 4.

Figure 3 shows the distribution of beliefs by treatment.^15^ When no message was sent (234_No), investors were more likely to believe that *k* would take low values, only 11% thought that it took the value of 4, while 50% thought it was 3 (the expected value). In those treatments where a message was sent (234_ExAnte and 234_ExPost), beliefs were even more pessimistic, as a larger fraction of individuals thought that *k* took the values 2 or 3. The effect of the message was then to induce investors to believe that returns were lower. In fact, in the treatment “234_ExAnte,” 63, 29, and 9% of investors thought that the value of *k* was 2, 3, and 4, respectively. This distribution is significantly different from the one corresponding to the treatment 234_No (Kruskal–Wallis test *p* = 0.0547). In the treatment “234_ExPost,” 53, 44, and 3% of investors believed that the value of *k* was 2, 3, and 4, respectively. In this case the difference with 234_No is not significant (Kruskal–Wallis test *p* = 0.1394), but the number of observations is lower.

The questionnaire responses suggest that investors expected false messages; in fact, they expected that the value of *k* would be overstated and upon receiving low values, found them believable. It is clear that the messages were not cheap talk for a sizeable proportion of subjects. This is important as it raises the possibility of strategic behavior both on the part of investees and investors. In addition, the introduction of messages made the investors more pessimistic about the value of *k*.

We now analyse whether messages are considered cheap talk by subjects in the treatment k>1_ExAnte. Results in this case are very similar to those found above. When we analyse whether investors actually believed the messages, we find that under ambiguity the proportion of investors who believe the message is still sizable (38%). Interestingly, 15% of the investors thought that the investees were under-reporting the value of *k*. This is larger than the proportion that believed that the message was understated by the investees in the 234_ExAnte treatment, 6.45%.

**Figure 4:**
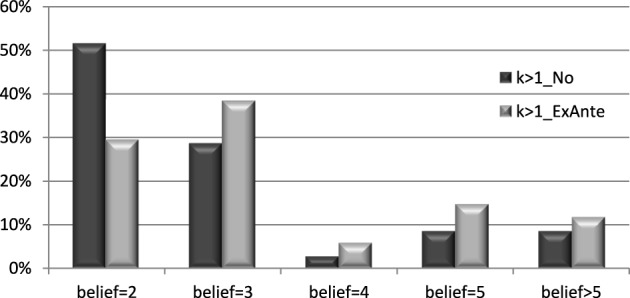
**Distribution of beliefs by treatment**. Ambiguity treatments.

As shown in Figure 4, in treatment *k* > 1_No, 51% of individuals thought that the value of *k* was 2, meanwhile 29, 3, 9, and 9% thought that it was 3, 4, 5, and >5, respectively. The effect of the message seems to be very different in this case as it moves the distribution of beliefs to the right. When a message was sent only 29% of the subjects thought that the value of *k* was 2, while 38, 6, 15, and 12% thought that it was 3, 4, 5, and >5, respectively. According to a Kruskal–Wallis test, the difference is significant at the 10% level (*p* = 0.0903). This also shows that individuals have pessimistic beliefs under ambiguity, and contrary to what we found before, the message reverses this pessimism^16^.

Comparing those who believed the message with those who did not believe it, those who believed had an average payoff of 82.43 DEX, while this was 80.63 DEX for those who did not believe the message. These two numbers are not statistically different, whether we use a two-sample *t*-test (*p* = 0.8154) or the Wilkinson–Mann–Whitney test (*p* = 0.8759). An equality of medians test also reaches the same conclusion (*p* = 0.89). In contrast, those who lied obtained higher returns in the experiment; they received on average 106.52 DEX, while those who told the truth received 75.125 DEX. The absolute difference is now larger, and a one-sided *t*-test would give a *p*-value of 0.0599, even if the other tests do not report a difference that is statistically significant (*p* > 0.1).

### Why are false messages sent?

In this section we explore the motives behind under, and over, statement of messages. Earlier (Figure 1) we saw that 20.8% of the investees understated the value of *k*, while 16.42% overstated it. Those who overstated *k* could have done it for strategic reasons, while those who understated it could have done it due to both guilt aversion and strategic reasons if they believed that exaggeration is not credible. In what follows we will investigate this further.

#### Strategic reasons

In the survey we asked the investees *how much they expected to receive from the investors* (given the message they had sent). We can use this question to understand whether investees who overstated the value of *k* did it to get the investor to send them a larger amount. The amount they expected to receive should be larger for those who overstated *k* than for those who told the truth and those who understated it.

**Figure 5:**
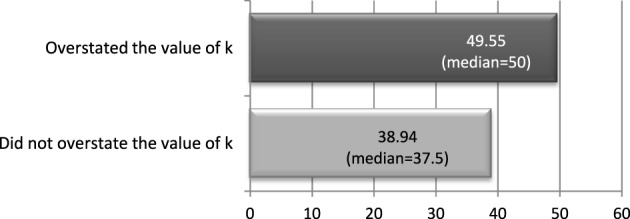
**Amount investees expected to receive from investors in treatment 234_ExAnte**.

Results using the treatment 234_ExAnte are shown in Figure 5. We find that subjects who overstated returns expected to receive on average 49.55 (median = 50), while the amount was 38.94 (median = 37.5) for subjects who told the truth or, understated the returns. A Wilcoxon–Mann–Whitney test shows that these two numbers are statistically different (*p* = 0.0997), in addition, a non-parametric test for differences in medians reaches the same conclusion (*p* = 0.033).^17^

Understatement can be due to strategic reasons as well. As we have seen above, messages with lower values of *k* were more believable, which is consistent with understating the value of *k* for strategic reasons, that is, to make the message more believable and induce the investor to send a larger amount. Also, we found a large percentage of investees that understated its value when *k* was 4.

#### Guilt aversion

Another reason for understating the value of *k* is to avoid anticipated guilt aversion. Some investees may feel guilty if they return a small amount knowing that the investor gets to know that *k* is large. If investees plan to return a small amount, then they may report a smaller *k* to avoid investor disappointment. If the investors could know the exact value of *k*, the message would stop being useful. To avoid investor disappointment, investees would then be more likely to return a larger amount than the amount they actually sent.

**Figure 6:**
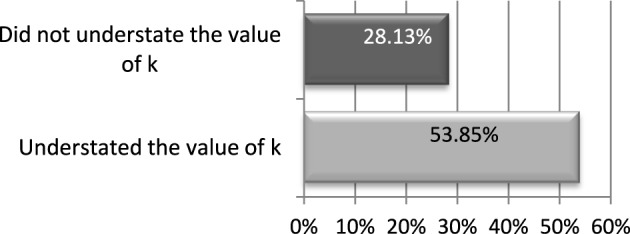
**Guilt aversion**. Proportion of investees that would have returned more if *k* was known. Treatment 234_ExAnte.

In the questionnaire we asked investees *how much they would have returned if the investor had known the exact value of k*. Figure 6 shows that out of those who understated *k*, 54% replied that they would have returned a larger amount if the investor had known the exact value of *k*. In contrast, out of those who did not understate the returns, just 28% replied that they would have returned more to the investor if the investor had known the exact value of *k*. The first proportion is significantly larger than the second one (*p* = 0.0512 in a one tail difference in proportions test).^18^

In the treatment 234_ExPost investees sent the message about the value of *k* ex post. The advantage of running the ex-post message treatment is that it removes the strategic motive to send a false message as ex-post messages have no impact on the amount sent.

**Figure 7:**
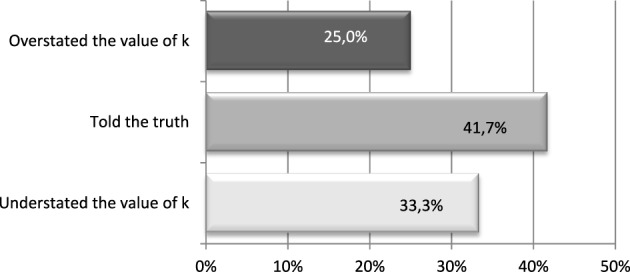
**Proportion of subjects who overstated the value of** ***k*****, understated it or told the truth**. Treatment 234_ExPost.

One can see from Figure 7 that 42% told the truth about the value of *k*, while the rest either overstated or understated its value. Out of those who overstated *k*, the majority (83%) expected to receive more from the investor than what they received. In contrast, the percentage is only 56% among those who did not overstate returns. We can think of these messages as inflicting some kind of non-monetary “punishment” on the investors. That is, the investee expected to receive more from the investor and when sending the message about the value of *k* reported a larger number than the true value. However, when comparing those who understated the value of the returns to the rest, approximately half the subjects of each group reported that they would have returned more if the investor had known the exact value of the returns.

### Questionnaire responses and false messages

We asked our subjects to respond to a questionnaire after the experiment and before the subjects knew their earnings. Through the questionnaire we can relate social and/or behavioral characteristics to subject choices in the experiment. Information was obtained on subject characteristics such as how frequently they lie to their parents, friends, acquaintances, and partners. Other questions asked them whether they lie to avoid harming other people or in their own benefit. They could answer using a Likert scale from 1 (very frequently) to 5 (never). We create dummy variables equal to one if the individual admits lying very frequently, frequently or sometimes, and zero otherwise. Below we elaborate on whether there is a significant relationship between these variables and subject behavior by running multinomial logit models in which we estimate the correlation between the answers to these survey questions and behavior in the experiment. Obviously, these variables are self-reported, but at least we can analyse whether those who sent false messages in the experiment about the value of *k* also admit lying in real life. Our dependent variable has 4 categories: uninformative message, lie understating, tell the truth, and lie overstating.

We first run a specification in which we analyse whether lying in the experiment is correlated to the subjects answering that they lie to their parents, friends, partner or acquaintances (results are reported in columns 1–4 of Table 4). Results in Panel A (without controls) and Panel B (with controls) show that those individuals who admit lying to their friends or partners are not more likely to send false messages in the experiment. Those who admit lying to their parents are more likely to send uninformative messages or to lie understating the value of *k*, however, these results become non-significant in the specifications in which we add control variables. Those who admit lying to acquaintances are more likely to tell the truth in the experiment, both in the specifications with and without controls. Control variables include a gender dummy, year of birth, a dummy for whether they are currently working, whether they are in a technical degree or in Sociology, Law, or Journalism (the reference category are Economics related degrees), and their average grade. We then analyse whether lying in the experiment is correlated to lying to avoid harming others or in their own benefit. Results are shown in columns 5–8 of both panels, with and without controls, respectively. Those subjects who claimed lying in their own benefit are more likely to tell the truth in the experiment, however the coefficient becomes non-significant when we add the controls. As before, individuals in the ambiguity treatment are both less likely to tell the truth and more likely to lie overstating the value of *k*.

**Table 4:** **False messages and questionnaire responses**.

**VARIABLES**	**1**	**2**	**3**	**4**	**5**	**6**	**7**	**8**
	**Uninformative message**	**Lie understating**	**Tell the truth**	**Lie overstating**	**Uninformative message**	**Lie understating**	**Tell the truth**	**Lie overstating**
**PANEL A: WITHOUT CONTROLS**										
Lies to parents	−0.135^*^	0.194^**^	−0.0212	−0.0386						
	(0.0742)	(0.0952)	(0.0825)	(0.0932)						
Lies to friends	0.103	0.0108	−0.109	−0.0054						
	(0.142)	(0.132)	(0.103)	(0.135)						
Lies to partner	0.0645	0.229	−0.135	−0.158						
	(0.165)	(0.205)	(0.124)	(0.142)						
Lies to acquaintances	−0.0678	−0.0481	0.175^*^	−0.059						
	(0.0814)	(0.0869)	(0.093)	(0.0914)						
Lies to avoid harming others					−0.0351	0.0109	−0.0385	0.0628
					(0.0811)	(0.0796)	(0.0869)	(0.0845)
Lies in own benefit					−0.122	−0.0547	0.211^*^	−0.0341
					(0.0861)	(0.0918)	(0.116)	(0.0993)
**PANEL B: WITH CONTROLS**										
Lies to parents	−0.113	0.137	0.0101	−0.0341						
	(0.0831)	(0.107)	(0.092)	(0.104)						
Lies to friends	0.0795	0.108	−0.127	−0.0611						
	(0.143)	(0.167)	(0.0974)	(0.124)						
Lies to partner	0.0148	0.329	−0.153	−0.19						
	(0.162)	(0.232)	(0.110)	(0.131)						
Lies to acquaintances	−0.0637	−0.0712	0.175^*^	−0.0401						
	(0.0912)	(0.0936)	(0.106)	(0.101)						
Lies to avoid harming others					−0.0268	0.0353	−0.104	0.0957
					(0.0848)	(0.0857)	(0.089)	(0.0919)
Lies in own benefit					−0.125	0.000692	0.193	−0.0696
					(0.0855)	(0.124)	(0.142)	(0.0996)
Treatment: k>1_ExAnte	−0.0599	0.0233	−0.192^**^	0.229^***^	−0.0523	0.0299	−0.209^**^	0.232^***^
	(0.081)	(0.0883)	(0.0828)	(0.0846)	(0.0791)	(0.0859)	(0.0844)	(0.0842)

*Robust standard errors in parentheses*.

*** *p < 0.01*,

** *p < 0.05*,

* *p < 0.1*.

*All regressions have 121 observations. The results reported are marginal effects. The dependent variable has 4 categories: uninformative message, lie understating, tell the truth, and lie overstating. Results are for treatmnets “234_AM” and “Kunknown_AM.” Control variables are described in the text*.

## Discussion

Our experiments show that lying is prevalent and involves a complex cognitive process. Individuals may have beliefs about which form of lying may be credible (i.e., understatement) or may elicit a certain response (i.e., overstatement). We further find that understatement is more believable than overstatement. Further, more than half the individuals believe the messages they receive, though, a priori they have no reason to believe them. The different types of lying we observe can be observed by guilt aversion and strategic lying. We observe a combination of both indicating that individuals have preferences over lying that are conditioned by their beliefs. We further find that beliefs of investors are conditioned by the degree of asymmetric information.

As found by others earlier (Gneezy, 2005; Kriss et al., 2013) we find a few truthful people in our experiments. Restricting the sample to those who sent an informative message 66% of the subjects sent false messages. The value of the multiplier (*k*) did not affect the probability of lying, but understatement of the multiplier was more likely when the multiplier was 4 than when it was 3. Interestingly, the incidence of false messages is much larger in the ambiguity treatment than in the uncertainty treatment.

We elicit beliefs regarding the message investors received. This allows us to see whether subjects are naive. Almost half the investors believe the message they receive. Further, messages stating a lower value of *k* are more believable while messages stating a higher value of *k* are less likely to be believed. We find that subjects have more pessimistic beliefs regarding *k* under ambiguity, and while the message makes beliefs about the multiplier more pessimistic when investors know the distribution of *k*, the opposite is true in the ambiguity treatment.

Subjects both over, and under, -state the returns on the investment. We find this to be consistent both with strategic lying (Ottaviani and Squintani, 2006) and with guilt aversion (Charness and Dufwenberg, 2006). Understatement can also be explained by the fact that investees may know that lower values of the returns are more believable.

Finally, results from the questionnaire conducted at the end of the experiment show that those who tell the truth in the experiment are also more likely to report lying to acquaintances in real life. We did not include questions that we could use in order to measure different personality traits in the questionnaire. These personality traits could also affect the propensity to deceive or to be prone to deception.

Our results have some consequences for the design of organizations. They suggest that a less information opens the door for deceptive acts. More research is needed, however, on what are the institutional arrangements that minimize deception and encourage trust facilitating exchanges.

### Conflict of interest statement

The authors declare that the research was conducted in the absence of any commercial or financial relationships that could be construed as a potential conflict of interest.

## Footnotes

^1^Fischbacher and Föllmi-Heusi (2013) study what they define as lies in disguise. In their design subjects face no threat of being caught and no material incentives to honesty exist in their experiments. They can only observe the distribution of lying behaviour and find that individuals either lie fully, partially, or are honest. They explain both full cheating and honesty through the model of lying aversion. However, this model is not able to explain partial lying.

^2^For example, Bloom Energy an energy startup sold their project as an “industry creator” that would eventually substitute the domestic grid.

^3^Evidence suggests that the economic loss to society can be quite large. For example, Mazar and Ariely (2006) state that the loss from returning used clothes to the fashion industry is estimated at $16 billion. The cost to the US economy from the Enron and WorldCom crisis was approximated at $37–42 billion.

^4^Kartik (2009) introduces the cost of lying in a model of strategic information transmission.

^5^Note that this explanation is similar to an understatement being more believable.

^6^There is a substantial literature on ambiguity in the Finance literature.

^7^In Sanchez-Pages and Vorsatz (2009), senders can choose whether to tell the truth, lie, or remain silent.

^8^Note that we didn't want to impose any constraint in the message sent by investees in treatment k>1_ExAnte. In order to keep treatments comparable, we didn't impose any constraints in treatments 234_ExAnte and 234_ExPost either.

^9^See Charness and Dufwenberg (2006, 2010).

^10^Note that this assumes that a player's utility depends on others' beliefs.

^11^Alternatively, those who send a message outside the distribution may have done it because they thought that any message sent may not be believable, or because they did not understand the game properly.

^12^Unfortunately, and due to software problems, we could only collect 50% of the answers to this question in the questionnaire.

^13^Three individuals reported a message outside of the distribution.

^14^In Figure S1 we plot our measure of trust (the investment), by the message received. We do this separately for the treatments 234_ExAnte and K>1_ExAnte. When the message receiver knows the distribution of *k*, pairwise tests show that investment is the same for the three types of messages. When the message receiver does not know the distribution, pairwise test show that investment when *k* is larger than 4 is greater than investment when it is exactly 4, however, it is not different from investment when *k* is 3. The other pairwise comparisons are not significantly different from zero.

^15^Restricting the sample to subjects who received informative messages and believed that *k* would take the values 2, 3, or 4.

^16^In Figures S2 and S3 we plot the distribution of beliefs by message received for both treatments in which a message is sent ex ante.

^17^A standard *t*-test would give a larger *p*-value, but the number of observations is too low to derive reliable conclusions.

^18^The two tails difference in proportions test gives *p* = 0.1025, however, the hypothesis we test in this case is whether the proportion who would return more is larger within those who understated *k* than within those who did not understate the value of *k*.
