# Supplementary material for: Information asymmetry and deception
Source: Front Behav Neurosci. 2015 Jul 21;9:109. doi: 10.3389/fnbeh.2015.00109 (PMC4508524; doi:10.3389/fnbeh.2015.00109)
Supplement: Supplementary file 1 [file DataSheet1.DOCX]

Supplementary Information

SI1 – Recruitment questionnaire.

This questionnaire was given to all subjects prior to their participation in the experiment.

1. In what year were you born?
2. Country of birth (or State in case of Spain).
3. Place of residence.
   - 1. Country or State
     2. City
4. If you were born outside of Spain (or outside your present State of residence) when did you arrive in your current residence (State)?
5. Country (or State for Spain) of birth of father.
6. Country (or State for Spain) of residence of father.
7. If your father was not born in Spain (or in the State where he currently resides) when did he arrive at his present residence?
8. Country (or State for Spain) of birth of mother.
9. Country (or State for Spain) of residence of mother.
10. If your mother was not born in Spain (or in the State where she currently resides) when did she arrive at his present residence?
11. Gender.
12. What is your major?
13. Year of study.
14. Average grade (0-10).
15. Religion.
16. Father´s studies (did not study/primary school/secondary school/university).
17. Mother´s studies (did not study/primary school/secondary school/university).
18. Total number of siblings (including you).
19. Do you have a partner (yes/no)?
20. Do you work? If yes, how many hours a week?
21. Do you do voluntary work? If yes, how many hours a week?
22. How many hours did you study (alone) last week?
23. How many hours of TV did you watch last week?
24. Are you a member of any political organization (yes/no)?

SI2 – Instructions

Sender instructions-*Part in italics vary according to treatments*

**No-Message (234_No):**

Thanks for coming. These instructions explain how the experiment works.

You have been selected at random as individual A. Another participant, in another place (outside of this room), has been selected at random to play with you. This person will be individual B. You and your partner will each receive 100 dex (experimental money).

After the experiment you will be able to convert the experimental money into real money that will be paid in cash at the end of the experiment. The exchange rate is 1 € = 12.5 dex.

This is how the experiment works:

*First, you will have the opportunity to transfer all, none, or part of the 100 dex to individual B. The amount sent to individual B will be multiplied by X, where X can take the values 2, 3 or 4 with equal probability, and is determined at random. The different values that X can take are also known for the individual B. Thus, if you send 50 dex and, for example, X = 3, individual B will receive 3x50 = 150 dex.*

*The amount you send will appear on the display of individual B, with the value that X has finally taken. Individual B will then have the possibility to send you back some of the amount received. Individual B may send any amount between zero and the amount you sent multiplied by X.*

*For example, if you send 50 dex to individual B and X = 3, then individual B receives 3x50 = 150 dex. Given this, individual B can send you any number between 0 and 150 dex. The amount individual B will send back will not be further multiplied.*

The experiment ends after the decision of individual B. Your earnings will be calculated on the following basis. You will be earning the initial 100 dex, minus the amount transferred to individual B, plus the amount individual B sends back to you.

Thus, if you send 50 dex to individual B and individual B sends back 70 dex, then your earnings will be 100 dex- 50 dex + 70dex = 120 dex. Applying the exchange rate this will be 9.60€. This will be your profit in this example.

The game will be played only once. Once the game ends, we will ask you to answer a few questions. Your answers will not have any influence on your earnings and will be treated as strictly confidential. The results of the experiment and the questionnaire will be used only in our research.

In the experiment today you will not interact with your partner again. You will not be able to know the identity of your partner. Similarly, your partner, nor any other participant, will know any details about you. Please do not talk to anyone during the experiment and raise your hand if you have any questions.

You are participating in a science experiment funded by the Ministry of Science and Technology. The information you give will not be associated with you and will be treated as confidential.

**Ex-ante message (234_ExAnte):**

*First, you will have the opportunity to transfer all, none or part of the 100 dex to individual B. The amount sent to individual B will be multiplied by X, where X can take the values 2, 3 or 4 with equal probability, and is determined at random. The different values that X can take are also known for the individual B.*

*Before you make the transfer to individual B, individual B knows the true value of X and may decide to send a message with information about the value of X. Note that individual B may choose to send you a true, or false, message, or not send a message at all.*

*Thus, if you send 50 dex and, for example, X = 3 (which does not have to match the value reported by the individual B), individual B will receive 3x50 = 150 dex.*

*The amount you send will appear on the display of individual B, with the value that X has finally taken. Individual B will then have the possibility to send you back some of the amount received. Individual B may send any amount between zero and the amount you sent multiplied by X.*

*For example, if you send 50 dex to individual B and X = 3, then individual B receives 3x50 = 150 dex. Given this, individual B can send you any number between 0 and 150 dex. The amount individual B will send back will not be further multiplied.*

**Ex-post message (234_ExPost):**

*First, you will have the opportunity to transfer all, none or part of the 100 dex to individual B. The amount sent to individual B will be multiplied by X, where X can take the values 2, 3 or 4 with equal probability, and is determined at random. The different values that X can take are also known for the individual B. Thus, if you send 50 dex and, for example, X = 3, individual B will receive 3x50 = 150 dex.*

*The amount you send will appear on the display of individual B, with the value that X has finally taken. Individual B will then have the possibility to send you back some of the amount received. Individual B may send any amount between zero and the amount you sent multiplied by X.*

*Once individual B decides the amount he will send to you, individual B may decide to send a message with information about the value of X. Note that individual B may decide to send a true, or false message, or not send a message at all. This message will be shown to you at the end of the experiment along with your earnings.*

*For example, if you send 50 dex to individual B and X = 3, then individual B receives 3x50 = 150 dex. Given this, individual B can send you any number between 0 and 150 dex. The amount individual B will send back will not be further multiplied.*

**k Unknown No Message (k>1_No):**

*First, you will have the opportunity to transfer all, none or part of the 100 dex to individual B. The amount sent to individual B will be multiplied by X (X>1). Individual B knows that X>1. Thus, if you send 50 dex and, for example, X = 3, individual B will receive 3x50 = 150 dex.*

*The amount you send will appear on the display of individual B, with the value that X has finally taken. Individual B will then have the possibility to send you back some of the amount received. Individual B may send any amount between zero and the amount you sent multiplied by X.*

*For example, if you send 50 dex to individual B and X = 3, then individual B receives 3x50 = 150 dex. Given this, individual B can send you any number between 0 and 150 dex. The amount individual B will send back will not be further multiplied.*

**k Unknown Ex-ante Message (k>1_ExAnte):**

*First, you will have the opportunity to transfer all, none or part of the 100 dex to individual B. The amount sent to individual B will be multiplied by X (X>1). Individual B knows that X>1.*

*Before you make the transfer to individual B, individual B knows the true value of X and may decide to send a message with information about the value of X. Note that individual B may choose to send you a true, or false, message, or not send a message at all.*

*Thus, if you send 50 dex and, for example, X = 3 (which does not have to match the value reported by the individual B), individual B will receive 3x50 = 150 dex.*

*The amount you send will appear on the display of individual B, with the value that X has finally taken. Individual B will then have the possibility to send you back some of the amount received. Individual B may send any amount between zero and the amount you sent multiplied by X.*

*For example, if you send 50 dex to individual B and X = 3, then individual B receives 3x50 = 150 dex. Given this, individual B can send you any number between 0 and 150 dex. The amount individual B will send back will not be further multiplied.*

Receiver instructions- *Part in italics vary according to treatments*

**No-Message (234_No):**

Thanks for coming. These instructions explain how the experiment works.

You have been selected at random as individual B. Another participant, in another place (outside of this room), has been selected at random to play with you. This person will be individual A. You and your partner will each receive 100 dex (experimental money).

After the experiment you will be able to convert the experimental money into real money that will be paid in cash at the end of the experiment. The exchange rate is 1 € = 12.5 dex.

The experiment works like this:

*First, individual A will have the opportunity to transfer, all, none, or part of their 100 dex to you. The amount sent by individual A will be multiplied by X, where X can take the values 2, 3, or 4 with equal probability, and it is determined at random. Thus, if individual A sends 50 dex and, for example, X=3, you will receive 3 x 50 = 150 dex.*

*The amount sent by individual A will appear on your screen. You will also get to know the value taken by X. This value is not known to individual A, but he or she knows that X can take the values 2, 3, or 4 with equal probability and is determined at random.*

*You will then have the possibility to send back some of the amount received. You may send any amount between zero, and the amount sent by individual A to you multiplied by X.*

*For example, if individual A sends 50 dex to you and X=3, then you will receive 3x50 = 150 dex. Given this, you can send to individual A any number between 0 and 150 dex. The amount you send back will not be further multiplied.*

The experiment ends after your decision. Your earnings will be calculated on the following basis. You will be earning the intitial 100 dex plus the difference between the amount received from individual A and the amount you send back (to individual A).

Thus, if individual A sends 50 dex to you and you send back 70 dex, then your earnings will be 100 dex + 150 dex – 70 dex = 180 dex. Applying the exchange rate this will be 14.40€. This will be your earnings in this example.

The game will be played only once. Once the game ends, we will ask you to answer a few questions. Your answers will not have any influence on your earnings and will be treated as strictly confidential. The results of the experiment and the questionnaire will be used only in our research.

In the experiment today you will not interact with your partner again. You will not be able to know the identity of your partner. Similarly, your partner, nor any other participant, will know any details about you. Please do not talk to anyone during the experiment and raise your hand if you have any questions.

You are participating in a science experiment funded by the Ministry of Science and Technology. The information you give will not be associated with you and will be treated as confidential.

**Ex-ante message (234_ExAnte):**

*First, individual A will have the opportunity to transfer, all, none, or part of their 100 dex to you. The amount sent by individual A will be multiplied by X, where X can take the values 2, 3, or 4 with equal probability, and it is determined at random. Thus, if individual A sends 50 dex and, for example, X=3, you will receive 3 x 50 = 150 dex.*

*Before individual A makes the transfer to you, you will know the true value of X and may decide to send a message with information about the value of X. Note that you may choose to send a true, or false, message, or not send a message at all to individual A. Individual A will get to see the message you send, or a message stating that you choose not to send a message.*

*The amount sent by individual A will appear on your screen, with the value that X has finally taken. This value is not known to individual A, but he or she knows that X can take the values 2, 3, or 4 with equal probability and is determined at random.*

*You will then have the possibility to send back some of the amount received. You may send any amount between zero, and the amount sent by individual A to you multiplied by X.*

*For example, if individual A sends 50 dex to you and X=3, then you will receive 3x50 = 150 dex. Given this, you can send to individual A any number between 0 and 150 dex. The amount you send back will not be further multiplied.*

**Ex-post message (234_ExPost):**

*First, individual A will have the opportunity to transfer, all, none, or part of their 100 dex to you. The amount sent by individual A will be multiplied by X, where X can take the values 2, 3, or 4 with equal probability, and it is determined at random. Thus, if individual A sends 50 dex and, for example, X=3, you will receive 3 x 50 = 150 dex.*

*The amount sent by individual A will appear on your screen, with the value that X has finally taken. This value is not known to individual A, but he or she knows that X can take the values 2, 3, or 4 with equal probability and is determined at random.*

*You will then have the possibility to send back some of the amount received. You may send any amount between zero, and the amount sent by individual A to you multiplied by X.*

*Once you decide the amount you will send to individual A, you may decide to send a message with information about the value of X. Note that you may decide to send a true, or false message, or not send a message at all. This message will be shown to individual A at the end of the experiment along with his or her earnings.*

*For example, if individual A sends 50 dex to you and X=3, then you will receive 3x50 = 150 dex. Given this, you can send to individual A any number between 0 and 150 dex. The amount you send back will not be further multiplied.*

**k Unknown No Message (k>1_No):**

*First, individual A will have the opportunity to transfer, all, none, or part of their 100 dex to you. The amount sent by individual A will be multiplied by X (X>1). Thus, if individual A sends 50 dex and, for example, X=3, you will receive 3 x 50 = 150 dex.*

*The amount sent by individual A will appear on your screen. You will also get to know the value taken by X. This value is not known to individual A, but he or she knows that X>1.*

*You will then have the possibility to send back some of the amount received. You may send any amount between zero, and the amount sent by individual A to you multiplied by X.*

*For example, if individual A sends 50 dex to you and X=3, then you will receive 3x50 = 150 dex. Given this, you can send to individual A any number between 0 and 150 dex. The amount you send back will not be further multiplied.*

**k Unknown Ex-ante Message (k>1_ExAnte):**

*First, individual A will have the opportunity to transfer, all, none, or part of their 100 dex to you. The amount sent by individual A will be multiplied by X (X>1). Thus, if individual A sends 50 dex and, for example, X=3, you will receive 3 x 50 = 150 dex.*

*Before individual A makes the transfer to you, you will know the true value of X and may decide to send a message with information about the value of X. Note that you may choose to send a true, or false, message, or not send a message at all to individual A. Individual A will get to see the message you send, or a message stating that you choose not to send a message.*

*The amount sent by individual A will appear on your screen, with the value that X has finally taken. This value is not known to individual A, but he or she knows that X>1.*

*You will then have the possibility to send back some of the amount received. You may send any amount between zero, and the amount sent by individual A to you multiplied by X.*

*For example, if individual A sends 50 dex to you and X=3, then you will receive 3x50 = 150 dex. Given this, you can send to individual A any number between 0 and 150 dex. The amount you send back will not be further multiplied.*

SI3 – Post questionnaire

The following questionnaire was completed by the subjects at the end of the experiment (senders didn’t know their earnings). Questions were common for senders and receivers, except those indicated in square brackets.

| Do you think that you responded with care to the questionnaire? |
| --- |
| 1. Yes; 2. Yes, but I could have been more careful; 3. I am not sure; 4. No |
| With what frequency do you lend money to your friends? |
| 1. More than once a week; 2. Once a week; 3. Once a month; 4. Once a year (or less than that); 5. Never |
| With what frequency do you do you leave personal objects (CDs, clothes, videos, etc.) with friends? |
| 1. More than once a week; 2. Once a week; 3. Once a month; 4. Once a year (or less than that); 5. Never |
| How many close friends do you have? |
|  |
| Would you leave your belongings in the university locker without locking it? |
| 1. Yes; 2. No |
| Overall, can one trust people? |
| 1. Yes; 2. No |
| Do you think a large majority of people will take advantage of you if they had the opportunity? |
| 1. Yes; 2. No |
| With what frequency do you lie to your parents? |
| 1. Very frequently; 2. Frequently; 3. Some times; 4. Few times; 5. Never |
| With what frequency do you lie to your friends? |
| 1. Very frequently; 2. Frequently; 3. Some times; 4. Few times; 5. Never |
| With what frequency do you lie to the people you know? |
| 1. Very frequently; 2. Frequently; 3. Some times; 4. Few times; 5. Never |
| With what frequency do you lie to your partner? |
| 1. Very frequently; 2. Frequently; 3. Some times; 4. Few times; 5. Never |
| Have you ever benefitted from someone’s (unknown to you) generosity? |
| 1. Yes; 2. No |
| With what frequency do you lie to someone not to hurt their feelings? |
| 1. Very frequently; 2. Frequently; 3. Some times; 4. Few times; 5. Never |
| With what frequency do you lie to someone for your own benefit? |
| 1. Very frequently; 2. Frequently; 3. Some times; 4. Few times; 5. Never |
| A person’s wealth should not depend upon the effort which they make. Do you agree with this statement? |
| 1. Very much agree; 2. Agree; 3. Agree somehow; 4. Do not agree; 5. Very much disagree |
| These days one cannot trust unknown people. Do you agree with this statement? |
| 1. Very much agree; 2. Agree; 3. Agree somehow; 4. Do not agree; 5. Very much disagree |
| You consider yourself to be a trustworthy individual. Do you agree with this statement? |
| 1. Very much agree; 2. Agree; 3. Agree somehow; 4. Do not agree; 5. Very much disagree |
| When dealing with strangers one should always be careful (before you trust them). Do you agree with this statement? |
| 1. Very much agree; 2. Agree; 3. Agree somehow; 4. Do not agree; 5. Very much disagree |
| The majority of students do not copy during the exam? Do you agree with this statement? |
| 1. Very much agree; 2. Agree; 3. Agree somehow; 4. Do not agree; 5. Very much disagree |
| Do you trust other people when you do something of little importance with them? |
| 1. A lot; 2. Sufficiently; 3. A little; 4. Never |
| Do you trust other people when you do something of great importance with them? |
| 1. A lot; 2. Sufficiently; 3. A little; 4. Never |
| Do you trust your family? |
| 1. A lot; 2. Sufficiently; 3. A little; 4. Never |
| Do you trust your friends? |
| 1. A lot; 2. Sufficiently; 3. A little; 4. Never |
| Do you trust your neighbors? |
| 1. A lot; 2. Sufficiently; 3. A little; 4. Never |
| Do you trust your colleagues in the University? |
| 1. A lot; 2. Sufficiently; 3. A little; 4. Never |
| Can you trust the school system? |
| 1. A lot; 2. Sufficiently; 3. A little; 4. Never |
| Do you trust the police? |
| 1. A lot; 2. Sufficiently; 3. A little; 4. Never |
| Do you trust the judicial system? |
| 1. A lot; 2. Sufficiently; 3. A little; 4. Never |
| Do you trust the big corporations? |
| 1. A lot; 2. Sufficiently; 3. A little; 4. Never |
| Mr. Martinez is about to sell his car for 1,200 euros. The oil pump on his engine does not work well, and Mr. Martinez knows that if the buyer knew it he would have to reduce the price by 250 euros (the price of repair). If Mr. Martinez does not tell the buyer, the engine will overheat the first day of heat, causing damage worth 250 euros. Winter has just started and the buyer will not notice unless Mr. Martinez tells him. Mr. Martinez has decided not to mention this problem with the oil pump to the buyer. In your opinion, the behavior of Mr. Martinez is: |
| 1. Completely acceptable; 2. Acceptable; 3. Little acceptable; 4. Inacceptable |
| What would have been your answer regarding Mr. Martinez’s behavior if the cost of repairing the oil pump had been 1,000 euros (instead of 250 euros)? |
| 1. Completely acceptable; 2. Acceptable; 3. Little acceptable; 4. Inacceptable |
| [Senders] Considering the amount you sent, how much do you think that individual B will return to you? |
|  |
| [Receivers] How much did you expect individual A to send? |
|  |
| Think about some of your college friends who have more or less the same economic position as you. Imagine that your colleague has found 20 euros on the street. It is impossible to identify the owner and, therefore, it is completely acceptable and unquestionable from the moral point of view to keep it. Could you say that if you had found the money, this money would have been ______________ beneficial to you than to your colleague? |
| 1. much more; 2. more; 3. as much as; 4. less; 5. much less |
| [Senders - Baseline] If the amount sent would have been multiplied by an amount twice as much (than the actual amount), what amount would you have sent? |
|  |
| [Senders, all treatments except Baseline] With what value do you think the amount you have sent to individual B has been multiplied with? |
|  |
| [Senders, all treatments except Baseline] If you would have known that the exact value by which the amount sent was multiplied, 3 for example, how much would have sent of the 100 dex? |
|  |
| [Receivers, all treatments except Baseline] If individual A would have known the true value by which the amount is multiplied, what quantity would you have sent (as individual B) to individual A? |
|  |
| [Receivers, treatments 234_ExAnte, 234_ExPost and k>1_ExAnte] Did you find sending a message to individual A useful? |
| 1. Yes; 2. No |

Figure S1. Average trust by message received in treatments 234_ExAnte and k>1_ExAnte.

Figure S2. Distribution of beliefs by message received in treatment 234_ExAnte.

Figure S3. Distribution of beliefs by message received in treatment k>1_ExAnte.
